# Effects of dioxins on animal spermatogenesis: A state-of-the-art review

**DOI:** 10.3389/frph.2022.1009090

**Published:** 2022-10-21

**Authors:** Walaa Faiad, Chadi Soukkarieh, Denis J. Murphy, Abdulsamie Hanano

**Affiliations:** ^1^ Department of Animal Biology, Faculty of Sciences, University of Damascus, Damascus, Syria; ^2^ School of Applied Sciences, University of South Wales, Wales, United Kingdom; ^3^ Department of Molecular Biology and Biotechnology, Atomic Energy Commission of Syria (AECS), Damascus, Syria

**Keywords:** spermatogenesis, dioxins, endocrine disrupter, male infertility, environmental polluants

## Abstract

The male reproductive system is especially affected by dioxins, a group of persistent environmental pollutants, resulting in irreversible abnormalities including effects on sexual function and fertility in adult males and possibly on the development of male offspring. The reproductive toxicity caused by dioxins is mostly mediated by an aryl hydrocarbon receptor (AhR). In animals, spermatogenesis is a highly sensitive and dynamic process that includes proliferation and maturation of germ cells. Spermatogenesis is subject to multiple endogenous and exogenous regulatory factors, including a wide range of environmental toxicants such as dioxins. This review discusses the toxicological effects of dioxins on spermatogenesis and their relevance to male infertility. After a detailed categorization of the environmental contaminants affecting the spermatogenesis, the exposure pathways and bioavailability of dioxins in animals was briefly reviewed. The effects of dioxins on spermatogenesis are then outlined in detail. The endocrine-disrupting effects of dioxins in animals and humans are discussed with a particular focus on their effects on the expression of spermatogenesis-related genes. Finally, the impacts of dioxins on the ratio of X and Y chromosomes, the status of serum sex hormones, the quality and fertility of sperm, and the transgenerational effects of dioxins on male reproduction are reviewed.

## Introduction

Spermatogenesis is a biological process that includes a complex set of cellular events consisting of main four stages, (i) the mitotic proliferation of spermatogonial cells; (ii) the meiotic division to produce four haploid, round spermatids; (iii) the transformation of round spermatids into elongated spermatids; and (iv) the release of mature sperm into the seminiferous tubular lumen, a process referred to as spermiation. The process leading to the transformation of a primordial germ cell into a mature sperm varies among species, for example, one cycle of spermatogenesis takes 35 days in the mouse and hamster, 50 days in the rat, 45–65 days in various nonhuman primates, and 70 days in humans (1).

**Figure 1:**
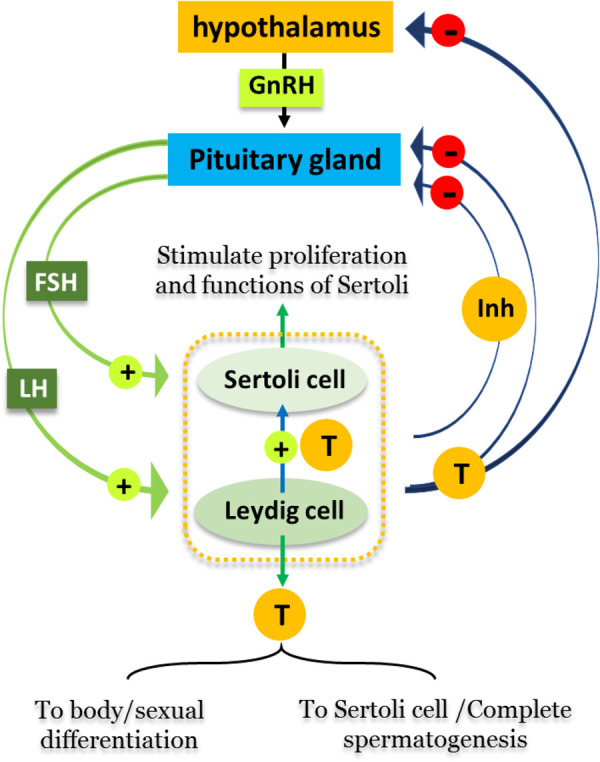
Hormonal regulation of spermatogenesis. Male reproductive cycles are controlled by interactions between secreted hormones from the anterior pituitary gland, hypothalamus and testis. The hypothalamus releases GnRH, hence stimulating the anterior pituitary gland to secret LH and FSH into the circulatory system. FSH stimulates Sertoli cells in the testis to begin spermatogenesis. Thus, when sperm counts increase Sertoli cells secrete inhibitors (Inh) into the blood that have a negative feedback on the hypothalamus, inhibiting the release of GnRH. LH stimulates Leydig cells to produce and secrete the hormone testosterone (**T**) into blood. When testosterone levels increase, it affects hypothalamus/pituitary gland by a negative feedback mechanism, thus inhibiting the production of LH, FSH, and GnRH.

Spermatogenesis is subjected to regulation by a group of hormones secreting *via* the hypothalamus-pituitary-gonadal (HPG) axis. Firstly, gonadotropin releasing hormone formed in the hypothalamus stimulates the anterior lobe of pituitary gland to produce Luteinizing hormone (LH) and Follicle-stimulating hormone (FSH). The binding of LH to its receptor on Leydig cells induces production of testosterone, which binds to the androgen receptors located on Sertoli cells, Leydig cells, and peritubular cells and therefore plays crucial roles in sexual differentiation and spermatogenesis. FSH binds to receptors that are exclusively present on Sertoli cells, stimulating their proliferation, maturation and other functions. Both FSH and testosterone can act either alone or in concert during different stages of spermatogenesis, confirming the vital roles of both Sertoli and Leydig cells in the completion of spermatogenesis (2). Figure 1 summarizes the hormonal regulation of spermatogenesis.

Sertoli cells play a fundamental role in the physical and metabolic support of germ cells (3, 4), by nourishing the developing cells, providing structural support and phagocytosis of abnormal germ cells, and releasing spermatids at spermiation (5). In addition, germ cells are involved in the production of several proteins (activin, inhibin) that maintain the release of pituitary hormones, thus indirectly controlling the mitotic activity of spermatogonia (6). Sertoli cells also protect the germ cells through their participation in formation of the blood-testis barrier (BTB), which prevents the destruction of germ cells by immune responses (7–9). Leydig cells are located within the interstitial tissue of testis and responsible for the production and secretion of the hormone testosterone (T), which plays a major role in the maintenance completion of spermatogenesis (10) and male secondary sex characteristics (11).

Spermatogenesis is influenced by multiple factors, including oxygen tension, temperature variation, radiation, and hormonal status. Of these, hormonal status is considered the most sensitive and important factor which is regulated through (testis) steroid hormones, hypothalamic and pituitary hormones. Many studies have reported that exposure to environmental pollutants, such as dioxins, benzo[a]pyrene, DTT, pesticides and heavy metals, causes disturbance of spermatogenesis by disruption of endocrine system. Supplementary Table S1 summarizes the toxicological effects of some environmental contaminants on spermatogenesis.

Nowadays, there is increasing public concern about reported increases in human infertility, that affect about 190 million people globally (12). Male infertility can be caused by multiple factors but it is frequently attributed to unhealthy lifestyles, including nutrition (13). In connection with this, exposure to environmental and occupational hazards are also thought to be responsible for the increasing incidence of infertility (14). This could be attributed to industrial and technical facilities releasing considerable quantities of gases and chemical compounds into the environment with proven toxicological impacts on human health in general and on the reproductive system in particular (15). As a result, many extraneous chemicals have been found to accumulate in human bodies (16), even more in the umbilical cord blood of infants (17). The group of chemical toxins that particularly disrupts the endocrine system is referred to as endocrine-disrupting chemicals (EDCs).

Collectively known as dioxins, polychlorinated dibenzo-p-dioxins (PCDDs) and polychlorinated dibenzofurans (PCDFs) act as endocrine disruptor chemicals (EDCs). Such halogenated chemicals are extremely lipophilic and highly persistent in the environment, accumulating and therefore readily transmitting through both human and animal food chains. Dioxins are eventually taken up by humans, principally *via* animal-origin fatty foods such as fish, meat and eggs (18), and also possibly *via* vegetable oils (19–22).

The toxicity of dioxins in mammals including humans, is well reported, and varies according to the health status and age of exposed animals, the concentration, the duration and the route of exposure (23, 24). In experimental studies on animals, oral exposure to 2,3,7,8-Tetrachlorodibenzo-p-dioxin (TCDD) led to loss of hair, reduction in body weight, and a weakened immune system (25). Reports from the International Agency for Research on Cancer (IARC) have confirmed the ability of dioxins to promote carcinogenesis in many organs such as skin, thyroid gland, liver, and the lymphatic system (26). These cancers are related to wasting syndrome (27), immunotoxicity due to the atrophy of some lymphoid and thymus tissues and to a decrease in the proportion of T cells and secreted cytokines, thus weakening the body's immunity defense against tumors (28), teratogenicity (29), alternation in genes expression related to lipid and glucose metabolism (30), dermal lesion including hair loss, hyperkeratosis, and chloracne (31–34), dysfunctional reproductive systems (35).

It is now well recognized that the reproductive toxicity of dioxins is mediated by the Aryl hydrocarbon receptor (AhR) pathway, and a transcription factor belonging to the basic helix-loop-helix (bHLH)-PAS gene family that acts as a receptor for many endogenous, including indigo, indirubin, 2-(1′H-indole-3′-carbonyl)-thiazole-4-carboxylic acid methyl ester (ITE) and certain arachidonic acid (36) and exogenous ligands, including polycyclic aromatic hydrocarbons and halogenated aromatic hydrocarbons (37, 38). AhR mediates many processes essential for maintaining cellular homeostasis, immunity, and responses to various stresses (39). Specifically for male reproductive health, some of these effects include hypospadias, cryptorchidism, testicular cancer, poor semen quality, negative effects on the prostate and change in sex ratio by decreasing the number of male births (40–42). In addition, animals exposed to dioxins have shown alterations in levels of sex hormones, reduction of sperm count, and increased rates of miscarriages (43, 44).

Here we review the current knowledge of the most significant effects of dioxins on male reproductive systems in mammals. The sources, exposure pathways and bioavailability of dioxins in mammals, plus the molecular mechanism by which AhR mediates the toxicity of dioxins on spermatogenesis are covered and the endocrine-disrupting effects of dioxins in animals and human and their effects on the ratio of X and Y chromosome-bearing are discussed.

## Definition, properties and sources of dioxins

Dioxins, polychlorinated dibenzodioxins (PCDDs) and polychlorinated dibenzofurans (PCDFs), are the most toxic group of environmental pollutants that have been described to date (45). Chemically, dioxins consist of two aromatic rings linked *via* either one or two atoms of oxygen, and give rise, respectively, to PCDFs or PCDDs. This extremely stable structure contains one to eight positions that can be chlorinated, which confers both high structural stability and extreme hydrophobicity. Depending on the number and position of chlorination (*P* = 1–8), the dioxin group includes 75 PCDD and 135 PCDF congeners that vary significantly in terms of their overall toxicity (46, 47). The number and position of chlorines in dioxin molecules affect their toxicity by modifying their shape, determining thus their binding ability to the AhR receptor (48). Typically, congeners with chlorine atoms substituted in the lateral 2, 3, 7 and 8 positions of the aromatic rings are considered as the most toxic. Of these, 2,3,7,8-tetrachlorodibenzo-*p*-dioxin (TCDD), with a toxic equivalency factor (TEF) of 1.0, is the most toxic congener of all the dioxins (49, 50). Table 1 presents names, structure and Toxicity Equivalency Factors (TEFs) of the most potent congeners of dioxins. Atmospheric dioxins exist in both gaseous or solid-bound phases depending on the prevailing temperature degree (51). At high temperatures, the less chlorinated dioxin congeners tend to be found free in the vapor phase, while they are more likely found bound to other molecules at lower temperatures (52–57).

**Table 1:** Names, structure and toxicity equivalency factors (TEFs) of the most toxic PCDDs, PCDFs and PCBs.

TEF	Structure	Congener
Group 1: polychlorinated dibenzo-*p*-dioxins (PCDD)		
2,3,7,8-TCDD	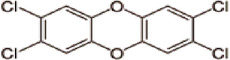	1
1,2,3,7,8-PeCDD	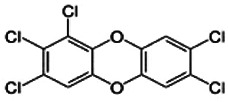	1
1,2,3,4,7,8-HxCDD	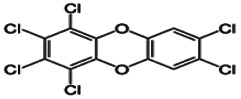	0.1
1,2,3,6,7,8-HxCDD	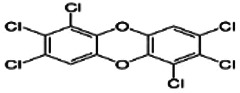	0.1
1,2,3,7,8,9-HxCDD	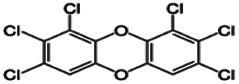	0.1
1,2,3,4,6,7,8-HpCDD	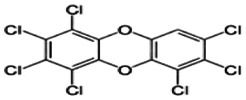	0.01
1,2,3,4,5,6,7,8-OCDD	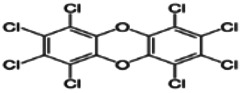	0.0001
Group 2: polychlorinated dibenzofurans (PCDF)		
2,3,7,8-TCDF	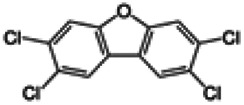	0.1
1,2,3,7,8-PeCDF	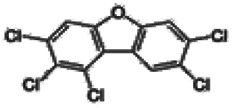	0.05
2,3,4,7,8-PeCDF	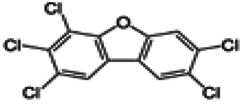	0.5
1,2,3,4,7,8-HxCDF	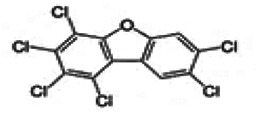	0.1
1,2,3,6,7,8-HxCDF	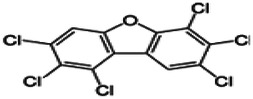	0.1
2,3,4,6,7,8-HxCDF	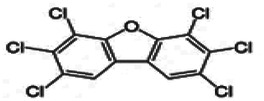	0.1
1,2,3,4,6,7,8-HpCDF	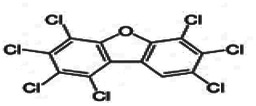	0.01
1,2,3,4,7,8,9-HpCDF	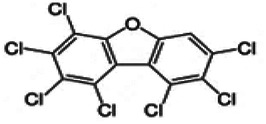	0.01
OCDF	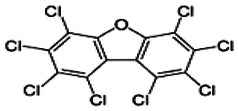	0.0001
Group 3: polychlorinated biphenyls (PCB)		
2,3,3′,4,4′-PeCB (105)	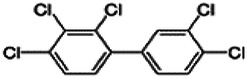	Mono-*ortho* PCBs ranged from 0.00001 to 0.0005
2,3,4,4′,5-PeCB (114)	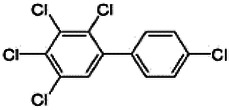	
2,3′,4,4′,5-PeCB (118)	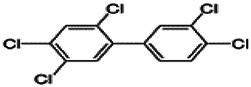	
2′,3,4,4′,5-PeCB (123)	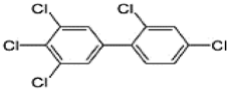	
2,3,3′,4,4′,5-HxCB (156)	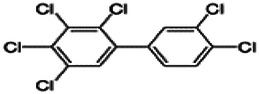	
2,3,3′,4,4′,5′-HxCB (157)	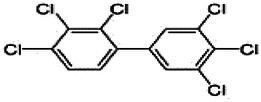	
2,3′,4,4′,5,5′-HxCB (167)	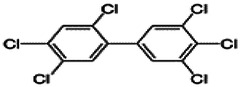	
2,3,3′,4,4′,5,5′-HpCB (189)	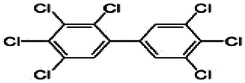	
3,3′,4,4′-TCB (77)	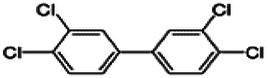	0.0001
3,4,4′,5-TCB (81)	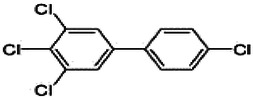	0.0001
3,3′,4,4′,5-PeCB (126)	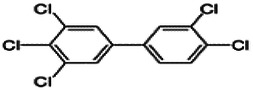	0.1
3,3′,4,4′,5,5′-HxCB (169)	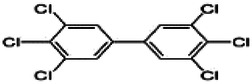	0.01

Dioxins are emitted into the environment from two main sources. The first one, which causes emission of only a small proportion of these chemical contaminants, is due to natural combustion-related process such as forest fires and volcanoes (58, 59). Over the last decade, the incidence of forest fires has increased resulting in higher dioxin releases from this source (60–62). Secondly, dioxins are released into the environment from industrial sources, such as processes involving melting of metals, production of PVC plastics, treatment of white paper pulp with chlorine, the combustion of coal, natural gas and petroleum products and wastes (63, 64), and the manufacture of chlorinated pesticides and herbicides for agriculture. The incineration of municipal and medical wastes as wastes from fossil fuels and fly ash storage are also important sources of dioxins (51). PCDD/Fs are also released from plastic bottles and containers exposed to microwave radiation or freezing (65), and from the vast and ever-increasing amounts of electronic wastes that are estimated to reach 52.2 million tonnes by 2021 (66). Ironically, recycling methods in processing of so-called e-waste, such as manual disassembly, roasting, acid leaching and open burning also result in the formation of PCDD/Fs (67–69). Finally, road transport emissions due to the combustion of carbon-based fuels are an important source of several halogenated organic pollutant, like PCDD/Fs, PCBs and polybrominated diphenyl ethers (PBDEs) (70–73).

## Exposure pathways and bioavailability of dioxins in animals

Although dioxin formation occurs at a local level in a given region, the eventual environmental distribution of these toxins can much wider. For example, once dioxins are released into the atmosphere, they bind to other particulates, even as small as PM_2.5_, such as incinerator ash or smoke and can remain suspended and windborne for lengthy periods before settling on terrestrial and/or aquatic surfaces at sites remote from their original release (74, 75). They can then be taken up by microbial organisms in aquatic environments and/or become attached to grasses, vegetables, and other crops (19–22, 76–79). Animals feeding on dioxin-contaminated grass such as cows, buffalo, goats, ducks, and chickens can concentrate dioxin in their tissues (especially in fat-storing liver and adipose tissues) so that the toxins move up through the food chain eventually entering humans *via* their diet (48, 80). The rate of absorption of dioxins depends on the route of exposure, their molecular size and solubility (81). For example, the absorption rate of TCDD through the small intestine and the lungs is about 50% and 90%, respectively (82, 83). This shows that the inhalation route for dioxins is more important that ingestion *via* food in such cases. Dermal absorption is much more limited, probably less than 1%, according to experimental studies on mice (81–83). Once dioxins are absorbed into the human body, they are readily distributed through the bloodstream to all organs and specifically tend to accumulate in liver and fatty tissues (84). In the liver, dioxins are converted to less toxic compounds that are water-soluble, but this detoxification process occurs very slowly. The rate of excretion and half-lives of dioxins differ among species, ranging from 11 days in hamster (84), 17–31 days in rats (81), and a remarkable 7–11 years in humans (84).

## Aryl hydrocarbon receptor (AhR)-mediating effects of dioxins on spermatogenesis

**Figure 2:**
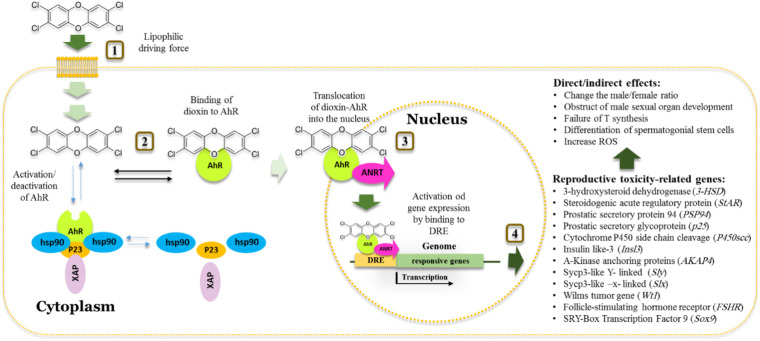
Schematic model of the action of dioxins in cell and aryl hydrocarbon receptor dependent toxic effect on Male reproductive system. **1**. Dioxins pass the membrane cell by a high lipophilic driving force. **2.** Once inside, the cell activates the aryl hydrocarbon receptor (AhR) receptor that normally found inactivated by complexing with Hsp90, XAP and P23 proteins. **3.** Binding of dioxin to AhR results in the release of other proteins. Translocation of dioxin/AhR into the nucleus occurred by dimerization of AhR with receptor nuclear translocator (ARNT). **4.** The AhR-ARNT heterodimer binds to dioxin responsive element (DRE), activating the expression of certain isoforms of *cyp1a* and *cyp1b* genes resulting in a range of activities including detoxification of dioxins and spermatogenesis-related disorders.

The AhR transcription factor has a strong affinity to a range of endogenous and exogenous ligands including polycyclic aromatic hydrocarbons and halogenated aromatic hydrocarbons (37, 38). AhR mediates many biological processes that are essential for maintaining cellular homeostasis, immunity, and responses to various stresses (39). Figure 2 summarizes the molecular mechanism by which AhR mediates the reproductive toxicity pathway in animals. In the dormant state, AhR is present in the cytosol as a heteromeric complex with two molecules of the 90 kDa heat shock protein (hsp90), and one molecule each of AhR-associated protein-9, and p23 (85). Binding of dioxin, the prototypical ligand, to AhR initiates series of events resulting in dissociation of hsp90, translocation into the nucleus and heterodimerization with an AhR-Nuclear Translocator (ARNT). Subsequently, the AhR-ARNT complex binds to specific DNA sequences, known as dioxin-response elements (DRE). In response to this activation, the AhR signaling pathway modifies expression levels of numerous genes predominantly encoding drug-metabolizing enzymes such as CYP1A1, growth factors such as EGF receptor and the estrogen receptor (ERs) (86).

In the respect of AhR roles in spermatogenesis, it is safe to say that there are multiple lines of biochemical and molecular evidence that confirm the vital roles of AhR in animal reproduction in both sexes. For example, AhR is actively expressed in the male reproductive tracts of mice (87, 88), and localized to germ and interstitial cells including round spermatids, elongating spermatids, and Leydig cells in the testis of mice (89). However, tissue differential expression of AhR was observed in the rat, where epididymis express AhR in a higher level than its level in the testis (90). In sharp contrast, the deletion of AhR is predominantly associated with defects in these functions, where the AhR-knockout mice had low sperm counts in the epididymis, a low fertility, a low level of serum testosterone and greatly reduced levels of steroidogenic 3-hydroxysteroid dehydrogenase (3-HSD) and steroidogenic acute regulatory protein (StAR) expression in testicular Leydig cells of *AhR* (–/–) males (87). In an indirect fashion, the activation of AhR by exogenous and/or endogenous ligands has been shown to have pronounced feedback on the various functions of reproductive system. AhR is therefore involved in mediating the toxicological effects of dioxin on the reproductive system. In this regard, a correlation between TCDD-activation of AhR and an alteration in the change of offspring sex ratio in mice was reported. Thus, wild type male mice exposed to TCDD had a 14% lower male:female sex ratio compared to AhR-knockout male mice that did not show any change in offspring sex ratio when exposed to the same dose of TCDD and their sex ratio was close to that of control mice (89).

More recently, histopathological studies showed that the exposure of male mice to dioxin-like compounds decreased the number of spermatogonia, sperm and Sertoli cells. Additionally, the percentage of testicular apoptotic cells was significantly raised, which was related to the downregulation of the GDNF/PI3K/AKT signaling pathway, suppressing the self-renewal and differentiation of spermatogonial stem cells (91, 92). Meanwhile, such exposure inhibited the genes expression of Sertoli cell markers (Fshr, WT1, Sox9) and the Leydig cell marker CYP11A1, impairing thus the function of Sertoli cells and Leydig cells (91). In line with this, it was reported that in mouse testicular TM4 Sertoli cells the tolerance to dioxin-like cytotoxicity is associated with insufficient AhR and CYP1A1 expression (93). Here, it is worthy to note that the ligand-activation of AhR and its subsequent consequences for reproductive system functions are largely dependent on the genetic background of individual (94). The large number of studies demonstrating the role of ligand-activation of AhR, mainly by TCDD, in the dysfunction of animal reproductively has led some to suggest that certain natural compounds could be used to prevent the anti-reproductive toxicity by targeting the AHR pathway (95).

Involvement of the AhR signaling pathway in the normal development of accessory sex organs including prostate and seminal vesicles has also been reported (96). The requirement of the AhR signaling pathway for normal accessory sex organ development such as prostate and seminal vesicles raises the question of the subsequent effects of AhR activation by dioxin on the development of such organs. In this context, it was shown that *in utero* and lactational exposure to TCDD of AhR wild type mice reduced the anterior, ventral and dorsolateral lobe weight of prostate and seminal vesicle. In addition, an alternation in gene expression of prostatic secretory protein 94 (PSP94) and a prostatic secretory glycoprotein (p25), both has androgen-dependent expression. In contrast, such defects were absent in AhR knockout *C57/BL6* mice exposed to TCDD (96, 97).

Activation of the AhR pathway by TCDD was mediated *via* an increase of mitochondrial reactive oxygen species (ROS) in many tissues including sexual organs (98, 99). While spermatozoa from AhR knockout mice are completely resistant to TCDD-induced loss of mitochondrial membrane potential, after either an *in vivo* or *in vitro* treatment, TCDD exposed WT mice showed increasing levels of ROS (100). However, contradictory results on humans showed no alteration in human sperm mitochondrial function following 24 h of *in vitro* exposure to similar and higher TCDD doses (101). These conflicting results could be explained by the relative high resistance of humans to dioxins compared with many other animals, including laboratory rodents (102). Also, a comparative set of data on the transcriptional levels between normal and abnormal semen of men showed that no correlations between the AhR and ARNT transcripts levels and sperm concentration, morphology and motility (103). In human, all seminiferous tubule stages of testes express AhR (104) that is localized in acrosomes that contain degradative enzymes (including hyaluronidase and acrosin) involving in the breakdown of the zona pellucida during fertilization (88). Alternatively, new molecular mechanism of TCDD's action in human Sertoli cells has been suggested. This was through interrogating the expression profile of small non-coding RNAs (sncRNAs), known also as microRNAs, that can modulate testicular function during spermatogenesis and that their altered expression may be factors involved in male infertility (105, 106).

## Endocrine-disrupting effects of dioxins in animals and humans

Dioxins are known as endocrine-disrupting chemicals (EDCs) that can interfere with hormonal systems and cause toxic effects on both male and female reproductive systems, developmental disorders and birth defects (107). Such chemicals are structurally similar to endogenous hormones and usually bind to their receptors and act through multiple mechanisms, such as mimicking endogenous hormones *via* an agonistic effect, blocking their action *via* an antagonistic effect, or interfering with their metabolic activity exerts estrogenic, anti-estrogenic and anti-androgenic activities, depending on dose and time of exposure (108).

The effects of TCDD on the male reproductive system are found whether due to the exposure of male occurs during adulthood or in the late fetal and early postnatal developmental periods. These effects vary depending on dose, duration of exposure (acute or chronic), and animal species. Experimentally, mice, rat, zebra fish, guinea pig, cock and monkey have shown diverse toxicological effects towards TCDD. For example, while a reduction in the size of testes and in the weight of sex glands (prostate, seminal vesicle) were reported in mice (96), a decrease in motility and count of sperm and an increase in the number of abnormal sperm in adult rats (109). In addition, *in utero-*exposure of the male rats to TCDD reduces epididymal and ejaculated sperm numbers (110). Furthermore, prenatal exposure to dioxins caused abnormalities of sperm with increased abnormal morphology, reduced motility, and reduced capacity to penetrate hamster oocytes (111). However, the histological studies showed a decrease of tubule diameter and maturation arrest at different stages of seminiferous tubule epithelium, increasing the intracellular spaces between Sertoli cells and germ cells and resulting in destruction the blood:testis barrier in TCDD exposed guinea pigs (112). Furthermore, a decreased intercellular contact in the germinal epithelium with Sertoli cells containing high levels of lipids, phagolysosomes, and vacuoles in their cytoplasm was observed in TCDD exposed marmoset (*Callithrix jacchus*) (113). Moreover, structural and functional damages in the cell membrane and necrosis of germ cells, disorders in contacts between Sertoli and spermatogenic cells, impairment of Sertoli cell function were detected in TCDD-exposed common marmosets (113–115).

In adult rats, TCDD reduces Leydig cell volume, leading to androgen deficiency (116), with a set of biochemical and molecular alterations including an increasing level of reactive oxygen species (ROS) and oxidative status, and affected antioxidant enzymatic activities, e.g., from *superoxide dismutase (SOD)* and catalase (CAT) (115). In contrast, adult rats exposed to high concentrations of TCDD showed reduced levels of circulating testosterone and dihydrotestosterone (DHT) (117). Also, TCDD-exposed rats have highly suppressed expression of glutamic acid decarboxylase 67, an enzyme involved in Gamma-Aminobutyric acid (GABA) synthesis in the brain, that potentially prevents the perinatal surges of LH and testosterone and compromising sperm counts (118). This was accompanied with a net reduction in ejaculated and epididymal sperm count, a lowering in sex accessory gland weights, and demasculinized and feminized morphology of prenatal TCDD exposed male rat offspring without a reduction in serum testosterone or androgen receptor (AR) levels (119). At the biochemical level, dioxin-exposed mice show alternations in spermatogenic markers such as acid phosphatase, alkaline phosphatase and lactate dehydrogenase (120). Similarly, the exposure of male chickens (cocks) to PCBs provoked a significant decrease in the testicular weight, serum testosterone level, damage in seminiferous tubule and reduce in the number of germ cells (121).

In line with effects of dioxins in animals, it was reported that semen quality in TCDD-exposed humans was seriously affected and this depended on the age at time of exposure. For example, boys aged of 1–9 years who were exposed to TCDD showed a decreased concentration of sperm in their adulthood, suggesting therefore that exposure to dioxin in infancy has critical effects on spermatogenesis (122–124). Also, adults who exposed to dioxin in infancy showed spermatozoa damage with reduced motility (125–127). Histological studies also revealed impaired steroidogenesis mediated by reducing the expression of certain steroidogenic markers such as StAR protein, 3-β-HSD, and 17-3-β-HSD. Further, maternal exposure to TCDD resulting in failure of testosterone production in Leydig cells of their adult male offspring, affecting therefore the testosterone-bound androgen receptor-mediated gene transcription which play a key role in spermatogenesis (128).

## Effects of dioxins on the expression of spermatogenesis related genes

It is now well recognized that the exposure of animals to dioxins causes alterations on transcriptomic level of multiple genes which contribute to vitamin and lipid metabolism, hormone synthesis and trafficking of molecules inside cells (92, 129). Exposure of zebrafish to TCDD alters the expression of apolipoprotein Bb (apobb) with a role in lipid transport, an uncoupling protein 1 (ucp1) that is involved in estrogen-stimulated cellular response, a steroidogenic acute regulatory protein (STAR) responsible for cholesterol and steroid synthesis, and the inhibition of DNA binding 2 (ID2) which regulates Sertoli cell function and meiotic cell divisions (129). Similarly, it was reported that the Leydig cells of TCDD-exposed rats showed reduction in gene expression of P450scc (CYP11A1), which mediates biosynthesis of progesterone and testosterone-related hormones (130). In line with this, TCDD-exposed male mice showed a significant decreases in expression levels of P450scc and LH receptor encoding genes (131). A similar increase was also reported for the aromatase gene that transforms testosterone to estradiol hormone in TCDD-exposed Sertoli cell (132). Additionally, TCDD-exposed rats showed changes in the expression of six testicular proteins including testis-speciﬁc heat shock protein 70 (Hsp70), protein disulfide isomerase A3 precursor, 3-phosphoglycerate dehydrogenase, non-muscle myosin heavy-chain type B-like protein, and superoxide dismutase 1 that were signiﬁcantly up-regulated, while the fertility protein SP22 and phosphatidylethanolamine-binding protein were down-regulated (133).

## Effect of dioxins on the ratio of X and Y chromosome-bearing live spermatozoa

Although equal numbers of X and Y spermatozoa are produced during spermatogenesis, environmental and occupational exposure to toxic pollutants can change the sex chromosome ratio in ejaculated spermatozoa. This can be reflected by an altered sex ratio at birth (SRB) and mostly occurs through the disruption of the hormonal system (134). The sex selection involves different events, from spermatogenesis and different Y:X sperm ratios before ejaculation, the differential behavior of Y and X sperm in the female reproductive tract, differential conception and implantation rates, and differential fetal loss. In normal situations, Y-bearing sperm have a higher chance of fertilizing an oocyte because they are smaller and more mobile than X-bearing sperm (135). However, environmental factors can change sperm characteristics, therefore remove the Y sperm advantage and this alone can alter SRB (41). Several studies have shown changes in the ratio of males to females at birth for human and animals in connection with environmental hazards, causing an effective reduce in the male/female ratio offspring (136, 137), suggesting therefore a possible decrease in the viability of Y spermatozoa than X (41, 134). These studies indicate that, in most cases, the altered male/female ratio offspring is coming from fathers who had been exposed to environmental hazards.

The effect of exposure to certain hazards was experimentally evaluated, and the ability of X spermatozoa to survive longer than Y spermatozoa under stressful conditions *in vitro* was reported (138). From these studies it was hypothesized that environmental pollutants can seriously affect sperm characteristics in terms of morphology, quantity, motility and Y:X ratio. It has been reported that paternal exposure to high levels of TCDD is associated with the birth of more females (139). Thus, TCDD concentrations in the serum of potentially exposed fathers were linked to a lower male/female sex ratio in their offspring and this effect can persist for several years after exposure (136, 137). Epidemiological studies suggest that exposure to TCDD can lower the male/female ratio of human offspring (113). One of the most important studies on humans was performed on local inhabitants who had been exposed to 2,3,7,8-tetrachlorodibenzo-p-dioxin (TCDD) as a result of chemical plant accident in Seveso, Italy in 1976, where the high TCDD exposure in both parents was significantly associated with an excess of female offspring (140, 137) Similar effects were reported in a study conducted on people who exposed to dioxins during herbicide and pesticide manufacturing in Russia and New Zealand, where a decrease of SRB was related to the paternal exposure to dioxins (137, 139, 141). Additionally, the *in vitro* exposure of human spermatozoa to TCDD affected sperm fertilization-associated abilities through their deleterious effects on motility, overall viability, capacitation, as well as differential viability of X and Y sperm (134). However, the effects of dioxin-like compounds, polychlorinated biphenyls (PCBs), on SRB showed conflicting results between exposed males and females. For example, men in Yucheng city in Taiwan who ingested PCB-contaminated cooking oils fathered fewer boys, but no effects were observed for exposed mothers (40). Contrary to this, no effect of PCBs on SRB was observed after a high exposure of people to PCBs during the Yusho disease incident in Kyushu, Japan (142). Furthermore, eating PCBs-contaminated fish lowered SRB for mothers (143), but increased SRB for fathers (144). Similarly, maternal exposure to PCBs was associated with lower SRB in a sample of San Francisco Bay area women (145). Otherwise, the SRB decreased when both parents were exposed to PCBs (146). In a comparative study on the impact of PCB contaminants on human sperm Y:X chromosome ratio between three European populations, Swedish, Polish and Ukrainian, and the Inuit population in Greenland, the authors found that the Greenlandic and Swedish men had higher Y:X ratio and higher levels of PCB-153 in blood than the Polish and Ukrainians, and the level of Y spermatozoa was positively correlated with PCB in the Swedish cohort, while a negative relationship was observed in the Polish cohort (147).

In mice, short-term exposure to TCDD affects the sperm motility and viability, and increases acrosome reaction. Interestingly, Y spermatozoa has lower survival times than X spermatozoa at high concentrations of TCDD resulting in a low fertilization rate (148). Furthermore, *in vitro* exposure of mouse spermatozoa to TCDD significantly decreased the fertilizing ability of Y-bearing spermatozoa and consequently reduced the proportion of embryos with XY chromosomes (148). Also, the *in vivo* exposure of mice to TCDD showed reduced the sex ratio of two-cell embryos (149), although the direct paternal or *in utero* exposure of rats to TCDD decreased the proportion of male offspring (149, 150).

A similar scenario was reported in zebrafish (151). Although many studies found that the lower SRB coincided with changes in sperm characteristics, e.g., morphological abnormality, reduced viability and motility and capacitation) (148), some of studies revealed that the abnormality of sperm characteristics had no effects on SRB (152). However, these inconsistent results could be due to several factors, e.g., type and duration of exposure, sample size, demographic characteristics of the exposed cohort and possibly the presence of other chemicals than those in question. We can conclude that exposure to TCDD affects the sex ratio, and this is likely mediated by a high level of testosterone/gonadotropin in men and a high level of estrogen in women, thus leading to production of more male offspring (153). In line with this, many reports identified dioxins as disruptors of sex hormonal systems, causing disorders in steroidogenic hormone synthesis and possibly a failure of testosterone synthesis. This critical point is reviewed in the next paragraph.

## Effects of dioxins on serum sex hormones

Reports on the effects of TCDD on hormonal levels are conflicting. Some studies show that the exposure of animals to TCDD led to a net decrease in LH and testosterone levels while other studies show no such changes. However, these contradictory data could be due to the dose and the route of exposure, the animal species, and its developmental stage as well as to how long after exposure the hormones were measured (115, 154, 155).. Regarding the dose, while exposure of rat female to a low dose of dioxin during pregnancy led to a reduction of testosterone in testes of offspring in the embryonic stage, exposure to high doses of dioxins led to reduced levels of LH hormones (156, 157). Regarding the route of exposure, Choi et al., demonstrated that injection of rat intraperitoneally with TCDD (50 µg/kg body weight) caused a significant decrease in serum testosterone levels and a significant increase in serum FSH, LH and estradiol levels (133). Regarding the animal species, adult male C57BL/6J mice that were intraperitoneally administered TCDD (100 µg/kg body weight) had reductions in intratesticular testosterone but no change in expression of LH hormone in the pituitary gland (131). Regarding the developmental stage, *in utero* and *lactational* exposure studies on rats have shown that gestational exposure to TCDD did not affect testosterone concentration in male rat offspring (119). This study conflicted with another study which reported 50% reduction of testosterone was observed in adult male offspring whose mothers had been exposed to TCDD during pregnancy and lactation (158), whereas circulating testosterone, DHT (dihydrotestosterone) decreased in adults rat exposed to TCDD (117). Chaffin et al*.* reported decreased circulating estrogen levels in female offspring of Holtzman rats exposed to 1 µg/kg of TCDD on gestational day GD15 (159). However, the results of this study conflicted with previous choi's study on TCDD exposed adult Sprague-Dawley rats (50 µg/kg) which reported an increase in estradiol levels, this disagreement can be explained by the difference of dose and exposure period and route of exposure.

## Effects of dioxins on sperm count, quality and fertility

Dioxins have proven deleterious effects on sperm characteristics such as their motility, quality, count and capacitation. Such effects were demonstrated in rats, where sperm quality and fertility were considerably reduced after exposure to TCDD (160). Exposure to TCDD also reduced sperm count and motility in the epididymis (161). Similarly, the exposure of Rhesus monkeys to TCDD in prenatal or in lactation periods led to reduction of sperm quality (162). In humans, it was reported that the high level of PCBs in the seminal plasma was correlated with a decrease of sperm motility (163). Similarly, a correlation between presence of PCB 126 in serum and viability in men with low semen quality was found (164). More recent results showed that TCDD exposure reduced the mice testis weight, sperm count and blood testosterone concentration, suggesting therefore that TCDD can cause damage to the male reproductive system of rodents through direct or indirect exposure (165).

## Transgenerational effects of dioxins on male reproduction

There are several lines of evidence confirming that the effects of TCDD exposure on animals and humans can persist into the next generation (166, 167). This was shown in humans exposed to TCDD as a result of the 1976 chemical explosion in Seveso, Italy, where a reduced motility and a low count of sperm of their male offspring during puberty was reported (122, 168). These effects are considered multi-generational when it is confined to TCDD-exposed adult males or the F1 generation of exposed mothers, or trans generational effects while it transmitted to F2 and F3 generations (169). In this context, it was reported that the ancestral exposure of Sprague-Dawley rats to TCDD (100 ng/kg BW/d) *via* intraperitoneal (IP) injections from gestational days 8–14 caused a net increase in the level of testosterone in 1-year-old Sprague-Dawley rats of the F3 generation (170). Similarly, it was found that TCDD promoted alterations in sperm morphology, lowered the level of serum testosterone and reduced the transit time of sperm in the epididymis in F1, F2 and F3 generations of male Wistar rats that were ancestrally exposed to TCDD (1 mg/kg) *via* oral gavage on gestational day 15 only (160). Similar ancestral consequences were showed in C57BL/6 mice following the exposure to TCDD (10 mg/kg) on embryonic day 15.5, where abnormalities in sperm morphology were observed as far as the F3 generation (171). More recently, male fertility assessment in adult rats, borne form adult female Wistar rats exposed to TCDD during the critical stage of organogenesis, revealed a significant decline in mating and fertility indices (124). Table 2 presents information on the models, species, dose, animal age, route of exposure, congeners used and the effects of dioxins on animals.

**Table 2:** Summarizes the information on the models, species, molecules used and effects of dioxins on animals.

Models	species	Dose/age	Compound	Route of exposure	Toxic effects	Reference
Zebrafish	AB strain	50 pg/ml at both 3 and 7 weeks post fertilization (during sexual differentiation and gonad maturation)	TCDD	waterborne TCDD	Decreased spermatozoa with concurrent increase in spermatogonia, and decreased germinal epithelium thickness, alter expression of genes associated with testis development, steroidogenesis, spermatogenesis, hormone metabolism and xenobiotic response	Baker et al. (2016)
Guninea pig		4–5 week old 1 µg/kg	TCDD	Intraperitoneal injection	Dissolution of germinal epithelium, disruption of tight junction between adjacent sertoli cell and altered germ cell at all developmental stages	Kim et al. (1999).
Hyline cocks		Ten-month-old Hyline cocks. 50 mg/kg for six consecutive weeks.	PCBs (Aroclor 1,254	Oral administration	Decreased testicular weight and serum testosterone, damage of the seminiferous tubules	Cai-qiao and Hui-li (2004)
Marmoset Monky	CALLITHRIX JACCHUS	mature marmosets 1 to 10 µg/kg	TCDD	Subcutaneous injection	Decreased intercellular contact in the germinal epithelium. The Sertoli’s cells exhibited an increased amount of lipids, phagolysosomes, and vacuoles in their cytoplasm. Leydig’s cells also exhibited a decrease in activity of 3*β*-hydroxysteroid dehydrogenase (3β-HSD) resulting in failure T synthesis	Rune et al. (1991)
Mice	C57BL6	8-week-old. PCB99 (10) and PCB153 (100)mg/kg	PCB99 and PCB153	Puberty/oral gavage	Showed a significant increase in Leydig cell apoptosis	Oskam et al. (2004)
Mice	C57BL6	Pregnant female on gestation day 13 (In utero and lactational exposure) 5 μg/kg	TCDD	In utero and lactational exposure/oral administration	reduced ventral, dorsolateral, and anterior lobe of prostate and seminal vesicle weight	Lin et al. (2002)
Mice	C57BL	Adult male (0.8 to 100 µg/kg)	TCDD	Intraperitoneal injection	reduction of P450scc and LHR in the testis which play essential role in T hormone	Mai et al. (2020)
Rat	Wistar	Maternal Exposure/ (0.5 to 2 µg/kg)	TCDD	maternal Exposure	decrease in reproductive organ weight, reduction in epididymal sperm reserves, percent motile and viable sperm with an increase in percent morphological abnormal sperm	Mai et al. (2020)
Rat	Albino	Adult male (90 days old) 27.5 µg/kg	TCDD	oral administration *via* gavage for four weeks	caused degeneration of seminiferous tubules, tubular necrosis, intratubular vacuolization, widened lumen and deshaped germ cells, induced testicular damage *via* creation disorders in oxidative stress parameters, serum hormone Level, and sperm parameters.	El-Gerbed et al. (2015)
Rat	Sprague–Dawley	Maternal Exposure (on gestational day 15)/(0.5–2 μg/kg)	PCB mixture Aroclor 1,221 (A1221):PCB138, PCB153, and PCB180	Prenatal exposure on gestational days 16 and 18,	Showed that prenatal exposure to PCB’s caused significantly altered gene expression of nine genes in the preoptic area of the hypothalamus, indicating that changes due to endocrine disruption are obvious as early as at birth.	Dickerson et al. (2011)
Rat	Sprague-Dawley	Maternal Exposure on GD15/ (0.064 to 1 µg/kg)	TCDD	oral dose/maternal Exposure	decreased of testis, seminal vesicle, and prostate weight, decreased sperm count, especially the epididymal sperm counts	Wilker et al. (1996)
RAT	Holtzman	50 pg/ml at both 3 and 7 weeks post fertilization (during sexual differentiation and gonad maturation)	TCDD	oral dose/perinatal Exposure on GD15	reduced anogenital distance, reduced weight of accessory sexual organs, reduced spermatogenesis, and caused remasculinization or feminization of male sexual behavior	Mably et al. (1992), Mably et al. (1992) and Mably et al. (1992)

## Conclusion and future prospects

It is recognized that dioxins represent a serious and persistent threat to the reproductive health of mankind and other living organisms. Animal experimental and human epidemiological studies have established that exposure to dioxins is associated with irreversible and transgenerational multiple adverse effects on male reproductive systems, which are mediated by AHR. These effects include hormonal disrupting, alteration in male/female ratios, production of poor semen quality, and obstructing the development of sexual glands. However, to further investigate these effects, it will be necessary to generate more comprehensive data at the molecular, cellular, and biochemical levels. Future studies should therefore focus on understanding the effects of dioxins on the cellular and molecular levels of germ cells, and the lesions in signaling pathways that are responsible for the dysfunction of spermatogenesis. Understanding the molecular mechanism of dioxins influence may enable researchers to develop novel treatments and strategies for the early prevention of the deleterious effects of dioxins on male fertility.
