# Supplementary material for: Effects of dioxins on animal spermatogenesis: A state-of-the-art review
Source: Front Reprod Health. 2022 Oct 21;4:1009090. doi: 10.3389/frph.2022.1009090 (PMC9634422; doi:10.3389/frph.2022.1009090)
Supplement: Supplementary file 1 [file Table1.docx]

**Supplementary information**

**Table S1** List of some environmental contaminants with toxic effects on spermatogenesis

| Reference | Toxic effects | Sources | Contaminant |
| --- | --- | --- | --- |
| [[176](file:///C:\Users\esme.johnson\Downloads\Manuscript.DOCX#_ENREF_176), [177](file:///C:\Users\esme.johnson\Downloads\Manuscript.DOCX#_ENREF_177)] | Decrease in sperm count, sperm DNA damage, impaired sperm motility, increase the reactive oxygen species status leading to oxidative stress, cause apoptosis of spermatozoa with ability to disrupt BTB (blood testis barrier) | Industrial sources, in food, dietary supplements, water, air, alcoholic drinks, tobacco | Heavy metals |
| [[178-180](file:///C:\Users\esme.johnson\Downloads\Manuscript.DOCX#_ENREF_178)] | Impaired Sertoli cell functions, alter sperm morphologies and reduce sperm count, produce free radicals and promote oxidative stress and epigenetic modifications in the cells, and lead to apoptosis in SSCs (spermatogonial stem cells) , decrease testosterone levels and testis weight, | Occupational, dietary and environmental exposure (fungicides, insecticides, herbicides) | Pesticides |
| [[181](file:///C:\Users\esme.johnson\Downloads\Manuscript.DOCX#_ENREF_181), [182](file:///C:\Users\esme.johnson\Downloads\Manuscript.DOCX#_ENREF_182)] | BPA can cause epigenetic effects leading to infertile of young children, decrease sperm count, sperm motility, morphology and DNA damage | Major component in dental sealants, lining material of food and beverage cans, baby bottles, thermal papers, compact discs, DVDs and other electronic circuits | Bisphenol A [BPA] |
| [[183-189](file:///C:\Users\esme.johnson\Downloads\Manuscript.DOCX#_ENREF_183)] | Decreased sperm count and sperm motility, germ cell apoptosis, epigenetic modifications of genes expression involved in spermatogenesis | Dermal exposure from rain coats, soap, shampoo, nail polish, fragrance bases for perfumery and cosmetic products | Phthalates |
| [[190](file:///C:\Users\esme.johnson\Downloads\Manuscript.DOCX#_ENREF_190), [191](file:///C:\Users\esme.johnson\Downloads\Manuscript.DOCX#_ENREF_191)] | Affect the microtubule assembly kinetics, change the number and length of microtubules and therein affect Sertoli cell functions, which finally disrupt germ cell differentiation, maturation, transport and completely disrupt spermatogenesis | Solvents are used for varied industrial applications | *n*-hexane |
| [[192](file:///C:\Users\esme.johnson\Downloads\Manuscript.DOCX#_ENREF_192)] | Significant reduction in both serum and intra-testicular testosterone levels, reduction in sperm quality in the epididymis. | Incomplete combustion of organic materials, e.g., diesel exhaust, cigarette smoke, industrial waste by-products | Benzo[a]pyrene |
